# Spatiotemporal Changes in Catch Composition of Marine Species Across Seawater Temperature Shift Points in Korean Water

**DOI:** 10.3390/ani15091212

**Published:** 2025-04-24

**Authors:** Hyithaek Ceong, Inyeong Kwon

**Affiliations:** 1 Department of Multimedia, Chonnam National University, Yeosu 58754, Republic of Korea; htceong@jnu.ac.kr; 2 Department of Smart Fisheries Resource Management, Chonnam National University, Yeosu 59626, Republic of Korea

**Keywords:** species diversity, species dominance, climate change, marine ecosystem, water temperature

## Abstract

This study aimed to evaluate the effects of water temperature and catch frequency on biodiversity and distribution in the waters around Korea. We analyzed spatiotemporal catch data reported by fishermen and evaluated the fluctuations in the dominantly caught species based on the timing of the water temperature changes between 1999 and 2021. This study found an upward trend in the water temperature, and species diversity increased in the East, West, and South Seas after the water temperature changed. Relatively stable species diversity was maintained in the East Sea/Sea of Japan, although variability increased significantly in the West and South Seas. The distribution in the caught species showed that *Octopodidae* spp. and *Ommastrephidae* spp. were increasingly caught more consistently alongside *Pleuronectidae* spp. Moreover, this dominance was maintained even after the change in water temperature and was shown to be relatively less sensitive to water temperature changes.

## Simple Summary

Climate change is causing ocean temperatures to rise, which affects the types and numbers of fish that can live in certain areas. This study looked at fishing data and water temperatures around Korea from 1999 to 2021 to understand how these changes impact marine life. The results show that as water temperatures increased, fish diversity changed across Korea’s three seas—the East, West, and South Seas. The East Sea remained relatively stable, but the West and South Seas experienced more noticeable changes in which species were caught. Some species, like octopus and squid, continued to be caught in high numbers and seemed less affected by temperature changes, suggesting that they are more adaptable. On the other hand, a valuable species, the Japanese flying squid, showed clear declines in catch numbers and changes in where it could be found. These findings suggest that better fishing rules, like limits on catch size and timing, are needed to protect fish populations. Also, creating marine protected areas that cover larger regions, especially across country borders, could help conserve fish species that move between areas and ensure the long-term health of the ocean ecosystem.

## 1. Introduction

The present concern regarding preserving biological diversity is partly based on the belief that biodiversity loss would lead to reduced ecosystem functions, affecting the numerous services they provide to society [1,2]. The role of biodiversity in marine ecosystems can be divided into three areas: functions contributing to the fundamental biological, chemical, and physical processes occurring within the ecosystem; services that the ecosystem provides to humans; and material resources that the ecosystem offers directly to humans, that is, ecosystem goods [3]. In particular, the areas in which biodiversity plays a large role (evaluated as high when assessed as low, medium, and high) are primary production, carbon flow, and nutrient cycling in ecosystem functions; disturbance regulation, remineralization, waste treatment, biological control, the monitoring of global change and ecosystem health, recreation tourism, and education in ecosystem services; and habitat and refuge, food resources, raw materials, genetic resources, and natural heritage in ecosystem goods [3]. Variations in species abundance, especially for those playing critical roles in nutrient flow, food web stability, or disturbance processes, can have profound impacts on ecosystem structure and function [4]. Biodiversity plays a critical functional role by enhancing the likelihood of including species that exert significant ecological influence and by promoting more efficient utilization of resources [4]. Differences in environmental sensitivity among functionally similar species stabilize ecosystem processes, whereas differences in sensitivity among functionally different species make ecosystems more vulnerable to change [4]. Simply, the higher the species diversity, the more stable the ecosystem function can be maintained due to increased functional substitution. When there is a difference in sensitivity between functionally similar species, even if one species declines in a specific environmental condition, another species can take over its dynamics, meaning the resistance to environmental change and the existence of various species within various functional groups increases the ability of the ecosystem to adapt to multiple environmental conditions. Current global environmental changes that affect species composition and diversity are therefore profoundly altering the biosphere functionality [4]. Multiple anthropogenic pressures, including unsustainable fishing, warming oceans, invasive species, and pollution, have collectively accelerated the global reduction in marine biodiversity, as seen in species extinction and ecological homogenization [5].

Various factors threaten marine biodiversity; in particular, fishery resources in areas where intensive fishing has continued extensively are more strongly affected by climate change, and fishery productivity in the waters surrounding the Northwest Pacific, including the East Sea, has decreased by 15–35% from 1930 to 2010 [6,7]. Highly productive areas are rapidly restructuring their marine ecosystems due to the synergistic effects of overfishing and climate change, meaning they cannot be restored through short-term management; thus, a new long-term fishery management approach that integrates environmental changes is required [7,8]. Furthermore, the traditional silo-structured management approach, which focuses on a single species or sector, is widely considered inadequate [1,9,10]. This approach has failed to shield marine ecosystems from human impacts [11] and prevent fishery stock collapses [12]. Consequently, these failures have significantly negatively affected the human communities reliant on these resources [13]. The inefficacy of conventional management systems and the need to restore and sustain ecosystem health have driven the call for change [10]. Alternatively, the need for ecosystem-based resource management that encompasses all non-resource factors, including climate change, has become increasingly apparent [14]. In particular, since the baseline setting of biodiversity goals among the items of ecosystem-based resource assessment is applied differently depending on the ecosystem to which it is used [7], it is necessary to understand the changing patterns of the target sea area of resource assessment. However, to understand the current biodiversity status and its conservation needs (including restoration and prevention), it is imperative to monitor fundamental parameters of structural and functional biodiversity [15].

Previously, many studies have been conducted to understand the diversity of species in marine ecosystems. Guisande et al. [16] developed a prediction model for species richness by considering variables such as water temperature, water depth, nutrient concentration, and area occupied to identify factors affecting the species richness of marine cartilaginous fish (sharks and rays). These results showed that water temperature had a greater effect on the species richness of sharks, and the area occupied had a greater impact on the species richness of rays [16]. In addition, Wernberg et al. [17] investigated the effects of climate change on the ecosystem in the southeastern and southwestern coasts of Australia and observed a decrease in important marine ecosystem plants, such as giant kelp (*Macrocystis pyrifera*), in the southeast sea area due to factors such as increased ocean temperature and decreased nutrients. Some species also showed an increase in population, with tropical herbivore (sea urchin) species expanding their ecosystem ranges [17]. When analyzing tropicalization using trawl data from the Bay of Somme (English Channel, France), a phenomenon was observed in which the proportion of fish species that feed in warm water increases due to ocean warming; moreover, a trend was observed toward a decrease in the number of fish species inhabiting cold water rather than tropicalization [18]. Based on these studies, it is judged that changes in the biodiversity of marine ecosystems are affected by factors such as water temperature, water depth, and nutrients. In particular, it can be seen that climate change promotes changes in the ecosystem of fish species, whereby changes in water temperature are significantly correlated.

The objective of this study is to assess the ecological effects of climate change on the marine ecosystem surrounding the Korean Peninsula by analyzing changes in species diversity and the spatiotemporal distribution of dominant catch species in relation to seawater temperature trends. To this end, we used 20 years of spatiotemporal catch data (June 1999–December 2021) reported by fishermen at 12 h intervals to calculate the Shannon–Wiener diversity index. We also applied time series analysis to seawater temperature data to identify changepoints, which served as reference markers for comparing shifts in species composition and dominance before and after temperature transitions.

## 2. Materials and Methods

### 2.1. Analysis Data

We used the spatiotemporal fishing activity data (from June 1999 to December 2021) maintained by the National Federation of Fisheries Cooperatives Safety Operation Headquarters. These are data obtained by fishermen reporting their fishing activities every 12 h after departure (Table 1). The CAR (Catching Activity Record) data do not disclose the fishing vessel number, which is personal information that can distinguish fishing activities. For these reasons, this study aimed to analyze spatiotemporal fishing patterns based on the names of the fish caught based on the temporal information of ‘fishing date’ and the spatial information of ‘large sea area’. The total number of records reported for 8248 days was 15,249,614.

To analyze the water temperature changes and fishing characteristics around the Korean Peninsula, we collected data on the temperature of offshore waters of Korea from the observed data (from June 1999 to December 2021) of the Serial Oceanography Investigation by the National Institute of Fisheries Science of Korea.

**Figure 1:**
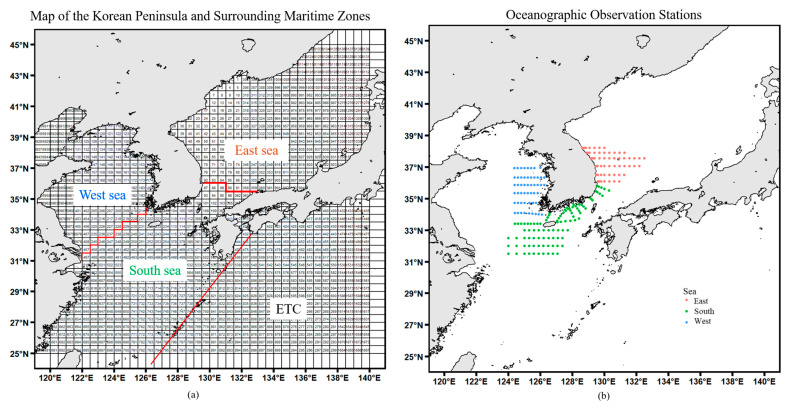
A map depicting the geographic divisions of the Korean Peninsula and surrounding maritime areas (**a**), along with the locations of oceanographic observation stations in each sea area (**b**).

To specifically distinguish the characteristics of fishing activity data and water temperature changes around the Korean Peninsula, the waters around the Korean Peninsula were divided into three seas without violating generality. The division between the West Sea and the South Sea was set by aligning a straight line connecting Jindo and Shanghai with the trench division, and the division between the East Sea and the South Sea was set by aligning a straight line connecting Mitsue, Japan, with the trench division. The KODC (Korea Oceanographic Data Center) data were divided close to the previously defined division to analyze the characteristics of water temperature change by sea area. The specific divisions are shown in Figure 1.

### 2.2. Analysis Method

**Figure 2:**
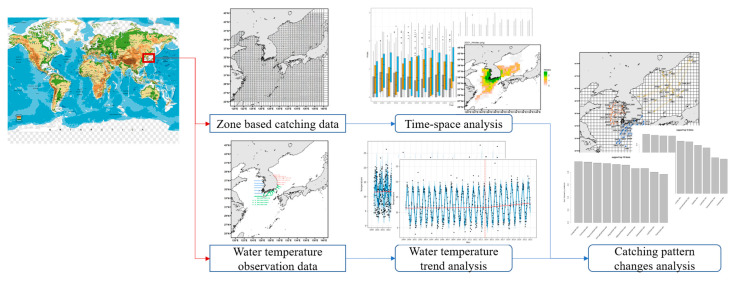
Analysis of catching patterns by species across geographical areas and time intervals in the Korean Peninsula.

The procedure for analyzing species diversity according to climate change in the waters around the Korean Peninsula is shown in Figure 2. Using reported catch data and observed water temperature data, a spatiotemporal analysis of species diversity was conducted, and trends in water temperature change were identified. In addition, an analysis of catching pattern changes was performed according to changes in water temperature and species diversity.

To analyze the spatiotemporal characteristics of fishing in the target sea area, the frequency of monthly and annual catches was analyzed using the data reported by fishing boats for each sea area, and the fishing characteristics were analyzed at the family level. To divide the sea area, a virtual line starting from Jindo, Jeollanam-do, and Shanghai, China, around the Korean Peninsula and a virtual line starting from Mitsue, Japan, in the Gyeongsangnam-do area were added as shown in Figure 1, and the East Sea was divided into 215 trenches, the South Sea into 233 trenches, the West Sea into 112 trenches, and other areas into 26 trenches. To organize the names of fish species (397) reported by fishermen, the Fisheries Production Trend Survey by Statistics Korea was used. WORMs were utilized to classify them at the family level for verification and correction. The final 3 kingdoms (Animalia, Plantae, and Chromista), 10 phyla, 20 classes, 72 orders, 143 families, and those not classified as species, such as ‘sea urchins’ or ‘jellyfish’, were classified as other. The Shannon–Wiener species diversity index (H′) [19] was used to analyze the spatiotemporal characteristics of the sea area based on the classified fish species.

(1) $$H-index=-\sum_{i=1}^{S}p_{i}ln(p_{i})$$

where H−index is the Shannon–Wiener species diversity index, S is the number of observed species (species richness), $p_{i}$ is the proportion of species i in the total sample, and ln is the natural log.

In calculating the species diversity index, the probability of species was calculated using the number of family names existing on a specific date as the frequency. This is because, due to the characteristics of CAR, no fishing boat number corresponding to the key exists to distinguish each record, meaning it is difficult to trust the catch amount. The species diversity index was calculated using family frequency with the date as the primary key. The calculated results were evaluated for the variability of species diversity (box plot width and whisker length) and diversity (IQR: interquartile range) through box plots.

We used the H-index to establish the criteria for population change in order to determine the spatial distribution of specific species (selected from commercially valuable species that have experienced a sharp decline or increase). Based on the selected species, catch per unit effort (CPUE) values were calculated for each fishing ground, allowing us to analyze spatial variation patterns over the years. CPUE was calculated by dividing the total catch (kg) by the number of fishing operations.

Various physical and chemical factors influence biodiversity, but considering water temperature as a key factor, fish species with more rapid generational turnover may exhibit the fastest demographic responses to water temperature changes [20], resulting in more pronounced distributional shifts in response to warming, which this study aims to analyze. To analyze the water temperature change in the waters around the Korean Peninsula and the characteristics and patterns of the caught species, the water temperature change was distinguished by sea area based on the previously distinguished virtual line. The prophet model was used to analyze trends, seasonality, and irregularities (holiday) through time series decomposition of water temperature changes [21].

(2) $$y\left(t\right)=g\left(t\right)+s\left(t\right)+h\left(t\right)+\epsilon_{i}$$

where g(t) is a factor for the time series trend, and piecewise regression or logistic growth curve was used. s(t) is a factor for the seasonality of the time series, while annual, weekly, and daily variability were analyzed using the Fourier series. h(t) is an irregular factor, which means it is a factor that is not explained by regular seasonality. $\epsilon_{i}$ is a random factor that is not explained by any factor. The change point in the water temperature trend was distinguished using the prophet model and identified as the point at which one can determine that the temperature trend changes over time. This study analyzed the change pattern of fish species, focusing on the change point. To distinguish the relationship between fishing characteristics and water temperature changes, the water temperature data were analyzed by applying the prophet model to the data from June 1999 to December 2022. We evaluated the Korean Peninsula and a univariate time series forecasting surface, mid-layer, and bottom water temperatures in each sea area.

For data analysis, R was used for data mining, and R packages (arules 1.7.7, arulesViz 1.5.2, [22]) were used for frequency items and visualization.

## 3. Results

### 3.1. Analysis of Fishing Spatiotemporal Characteristics

The changes in fishing frequency by year and month for the trench during the analysis period are shown in Table 2 and Table 3. The fishing frequency increased from 2011 to 2020 (daily average of 3712) (Table 2). However, the number of trenches where fishing occurred was, on average, 339 per year (minimum of 249 in 1999; maximum of 368 in 2016), showing no significant change (Table 2). The fishing frequency showed a decreasing trend in 2016 when fishing occurred in the largest number of trenches. Regarding monthly fishing characteristics, the highest amount of fishing occurred from July to October in the East Sea, while the fishing frequency decreased in the South Sea during that period. An analysis of the entire Korean Peninsula indicated that fishing activity was most active from July to November (Table 3). Catches in the Pacific Ocean off the coast of Japan are conducted in a small number of trenches (ETC) and are so infrequent that they were excluded from any subsequent analysis.

### 3.2. Analysis of Changes in Species Diversity

**Figure 3:**
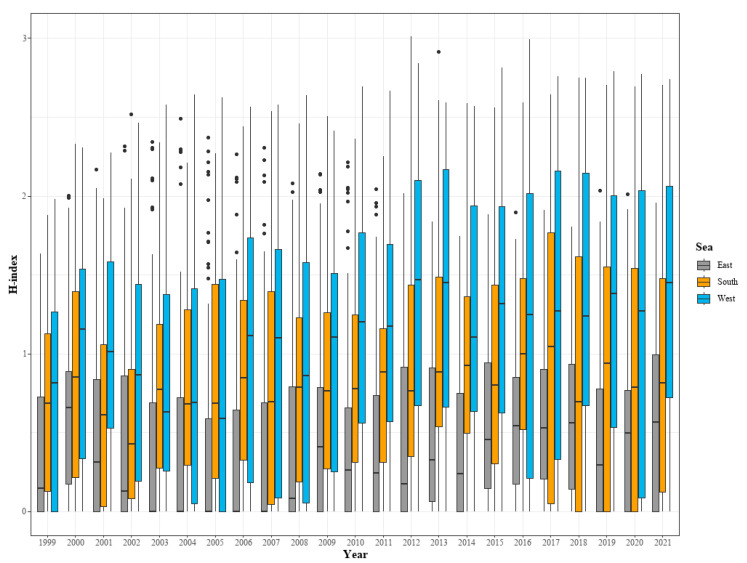
Changes in species diversity (H-index) by year and marine area from 1991 to 2021.

As a result of analyzing the species diversity (H-index) and the species caught in the East Sea, West Sea, and South Sea of Korea from 1999 to 2021, the H-index showed an upward trend in the analyzed seas overall (Figure 3). The East Sea showed a slight increase, but the IQR was the narrowest, maintaining a median value near 1 (Figure 3). Moreover, the length of the whiskers was relatively short compared to the other seas, indicating that the species diversity in the East Sea was relatively stable. In the West Sea, the IQR was wider than that of the other analyzed seas, and the median values were distributed between 1.5 and 2.5 (Figure 3). The length of the whiskers was longer than those for the other seas, indicating that the variability in species diversity was high (Figure 3). For the South Sea, the IQR was narrower than that for the West Sea but wider than that for the East Sea, and the median H-index was maintained between 1 and 2.

**Figure 4:**
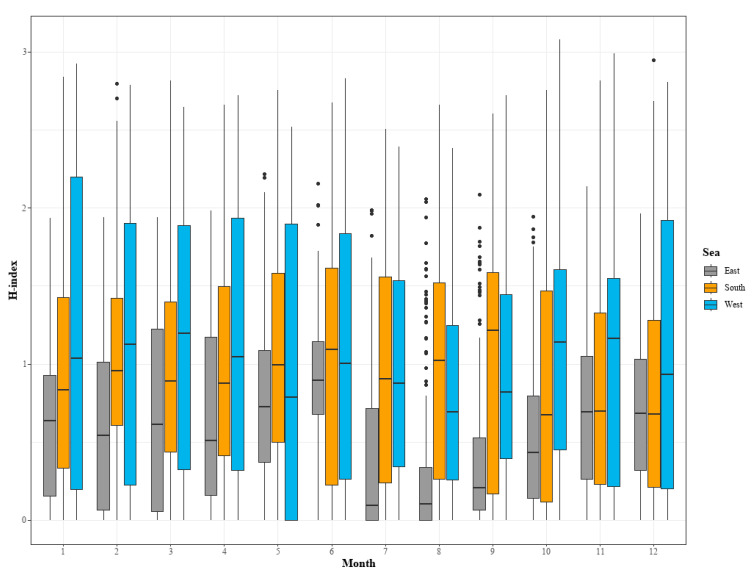
Changes in species diversity (H-index) by month and marine area in the Republic of Korea from 1991 to 2021.

In the West Sea, the median H-index was distributed between approximately 1.5 and 2.5, and it had the widest IQR in most months, showing that the variability of species diversity was greater than that in the other sea areas (Figure 4). In particular, many outliers appeared in July and at the end of September (Figure 4). In the South Sea, the median H-index was generally between 1.0 and 2.0, and the median was high in September and October (Figure 4).

**Figure 5:**
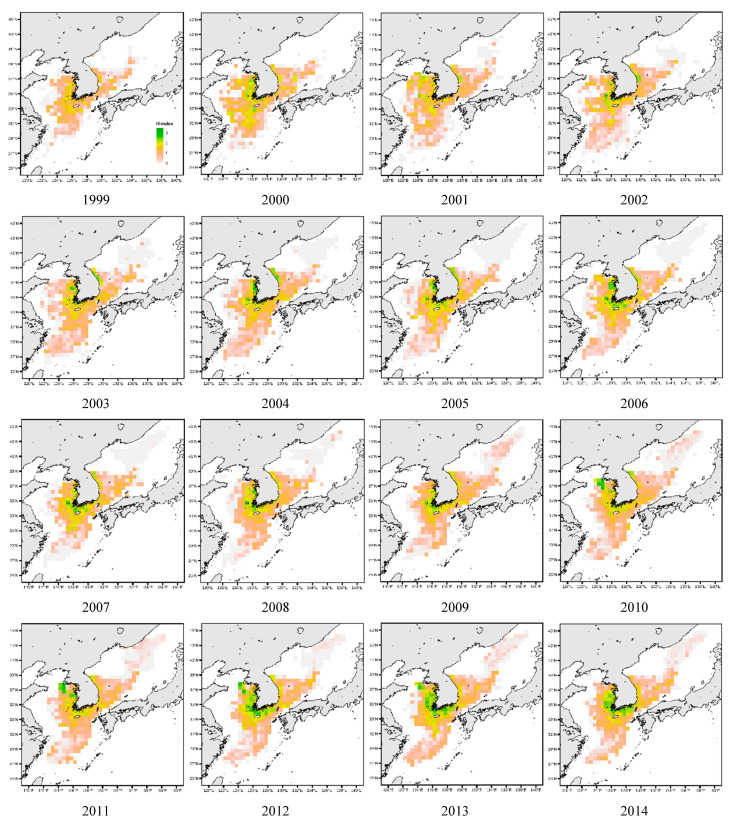

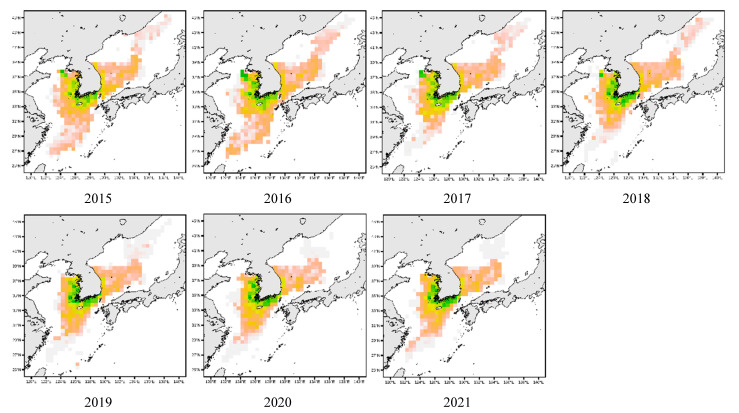
Changes in species diversity indices (H-index) in fishing zones by year.

Simply, a pattern of increasing species diversity was observed in the northeastern sea area (40°–42° N, 130°–132° E) with the H-index tending to increase slightly from the mid-2010s. This expansion was locally limited (Figure 5). In the southern area of the East Sea (35°–37° N, 129°–130° E), the green area began to decrease after 2010 and shrank significantly after 2015, promoting a rather distinct decrease in species diversity (Figure 5).

For the West Sea, a high species diversity (H-index) began to be observed in the central region; this range gradually expanded northward (near 38° N, 123°–126° E) and southward (near 32° N, 122°–124° E) from 2005 (Figure 5). In particular, the diffusion toward the north and south areas was noted as tending to expand from 2015 (Figure 5). In addition, spatial reduction in some areas of the West Sea was observed in the northern and southwestern waters, and a tendency for the H-index to decrease was observed near 37°–38° N and 122°–124° E. This area showed higher H-index values in earlier years, which appeared to decline after 2010. In the southwestern section, the green area decreased, and the H-index tended to decline in the southwest sea area at around 32°–34° N latitude and 122°–123° E longitude. High biodiversity was initially observed in this area; however, the reduction in green areas became more pronounced after 2010, becoming more evident after 2015.

Species diversity began to expand to the eastern and western parts of the South Sea after 2005 (Figure 5). In particular, the eastern waters (32°–34° N, 127°–129° E) showed increased biodiversity activities and an expansion in the H-index. In this region, species diversity activities increased post-2005, and its influence spread throughout the sea area. An expansion in the H-index was also observed in the western waters (33°–35° N, 125°–127° E); however, this expansion began relatively gradually before becoming prominent after the mid-2000s (Figure 5). A decreasing trend in biodiversity activities was observed in the East Sea around 33°–34° N and 128°–129° E. Moreover, the green area in this region gradually decreased alongside the H-index since 2010; this decrease has become more pronounced since 2015 (Figure 5). In the southwestern sea area, a decreasing trend in biodiversity was also observed around 31°–32° N and 125°–126° E. In this region, the decrease in biodiversity began after 2005 and has been more clearly observed since the mid-2010s. In particular, as the green area and H-index decreased, it was confirmed that the biodiversity in the southwestern sea area was decreasing. H-index values in the South Sea have increased in terms of spatial extent since 2005. However, spatial shrinkage has occurred in some seas since 2010, with the shrinkage range becoming clearer in 2015.

**Figure 6:**
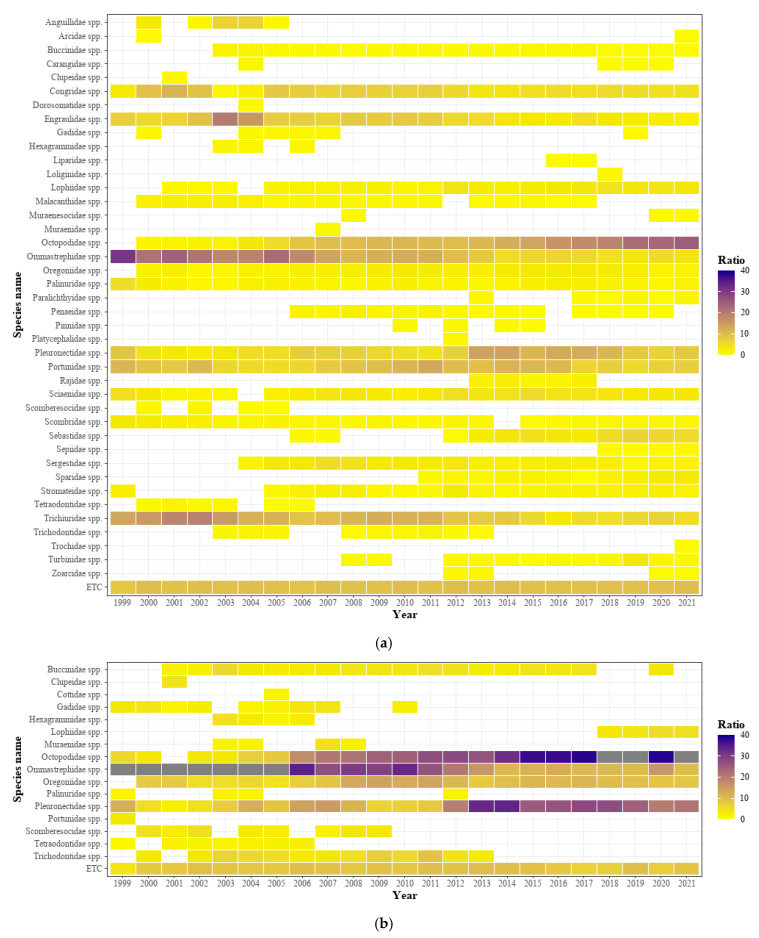

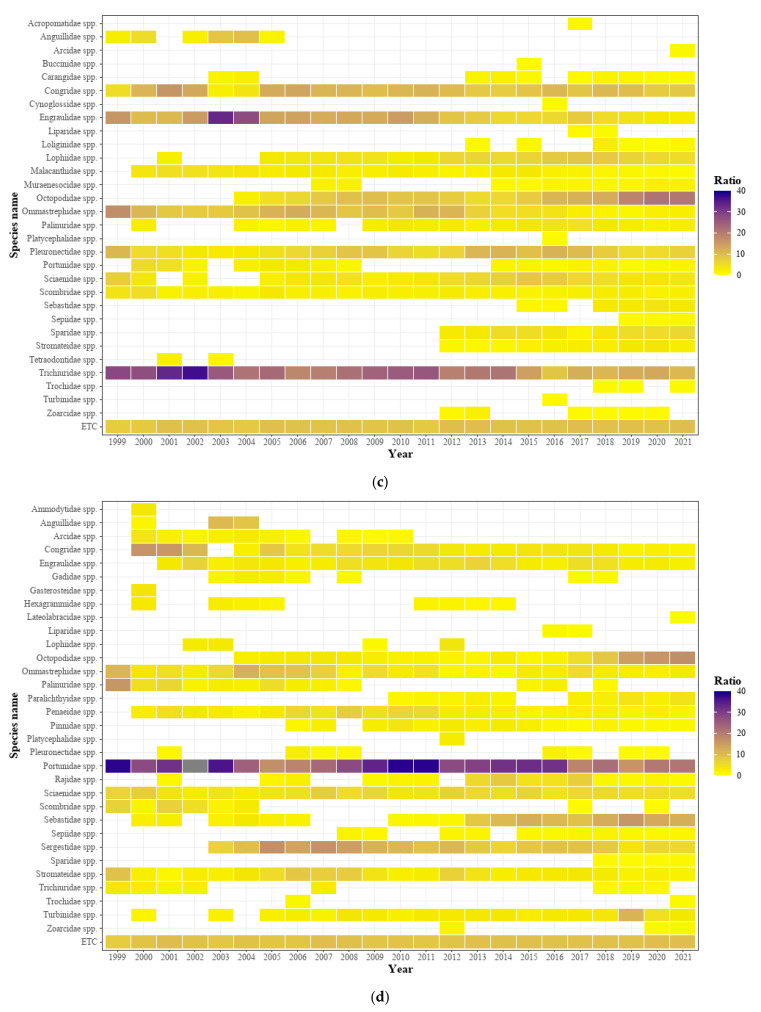
The proportion of key species, based on the frequency of catches in the maritime area in the Republic of Korea, account for over 90% of the total fishing data: (**a**) all seas, (**b**) the East Sea, (**c**) the South Sea, and (**d**) the West Sea.

Following the analysis of the major fish species caught in the waters surrounding Korea from 1999 to 2021, the dominant species in the entire waters was noted as *Octopodidae* spp., which has demonstrated a steady increase since 2004 (Figure 6a). Conversely, the opposite trend was shown by *Ommastrephidae* spp., which accounted for approximately 30–40% of the total catches between 1999 and 2006, but showed a gradual decrease (Figure 6a). In addition, the species that were caught previously but are currently rarely caught are *Clupeidae* spp., *Gadidae* spp., *Hexagrammidae* spp., and *Trichodontidae* spp.; meanwhile, the species that were not previously dominant but are presently gradually entering the catch ratio ranking are *Sebastidae* spp., *Sepiidae* spp., and *Sparidae* spp. (Figure 6a).

In the East Sea, *Octopodidae* spp. and *Pleuronectidae* spp. were the species that exhibited an increasing catch ratio. In contrast, *Clupeidae* spp., *Gadidae* spp., *Hexagrammidae* spp., *Trichodontidae* spp., and *Scomberesocidae* spp. were found to have been rarely caught recently (Figure 6b). In the South Sea, *Octopodidae* spp. showed an increasing catch ratio until recently, and although the catch ratio in each sea area varied by year, *Congridae* spp., *Engraulidae* spp., *Trichiuridae* spp., and *Pleuronectidae* spp. maintained a relatively constant dominant ratio (Figure 6c). In the West Sea, there was an increase and decrease by year, but *Portunidae* spp. had the highest catch rate, and *Sebastidae* spp. and *Ocotopodidae* spp. had high catch rates over the past 5 years (Figure 6d). In addition, species that were caught previously but have not been caught recently included *Arcidae* spp. and *Hexagrammidae* spp. (Figure 6d).

### 3.3. Analysis of Dominant Species by Changes in Water Temperature

**Figure 7:**
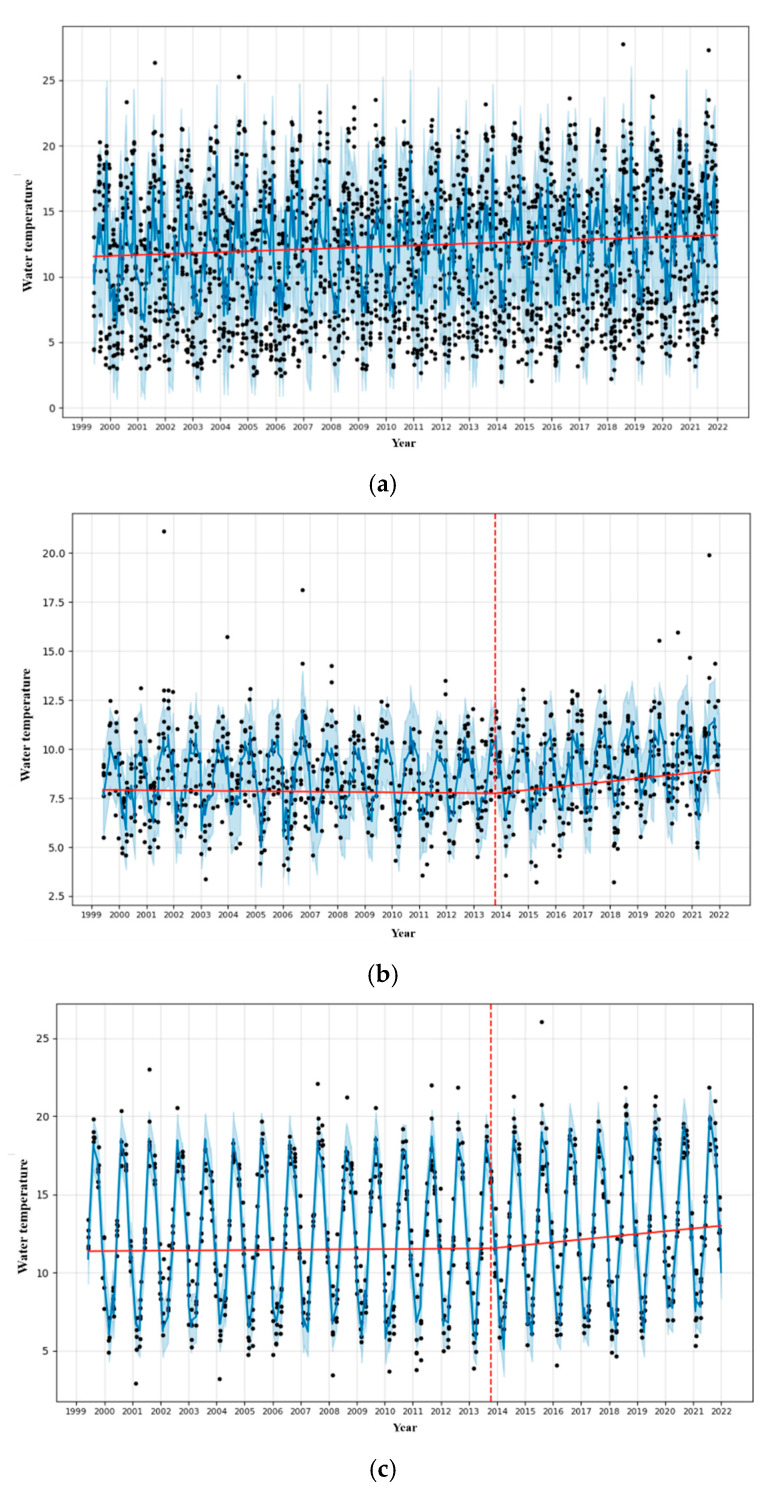

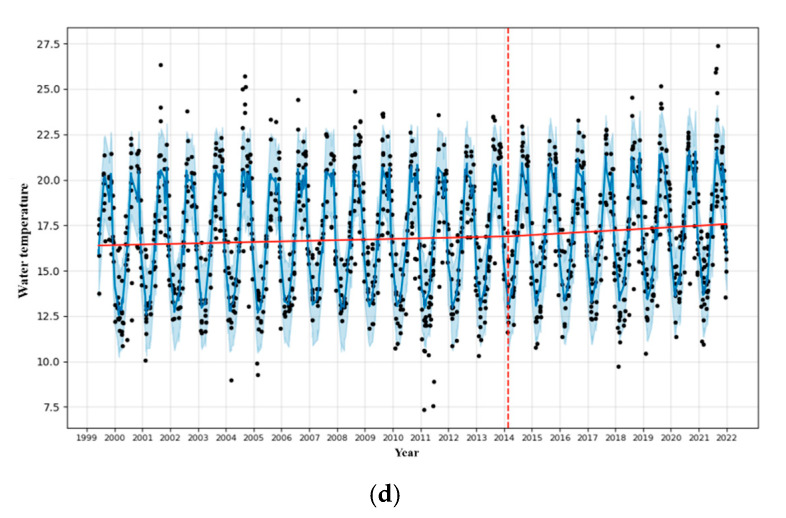
The results of the univariate time series analysis for surface and bottom water temperatures around the Korean Peninsula; the red vertical dashed lines indicate the points of temperature change: (**a**) all seas, (**b**) the East Sea, (**c**) the South Sea, and (**d**) the West Sea.

The changes in waters around the Korean Peninsula showed a trend toward rising water temperatures, as shown in Figure 7a. However, there was no point in which changes occurred before or after a specific time. Next, the characteristics of these water temperature changes and change points by sea area were distinguished. The East Sea changed on 20 October 2013, the West Sea on 15 October 2013, and the South Sea on 25 February 2014 (Figure 7b,c).

**Figure 8:**
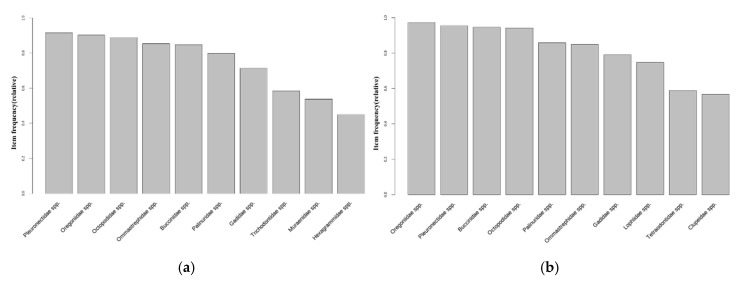
The frequency in which the top 10 species were caught in the transactions in the East Sea: (**a**) before the water temperature changing point (20 October 2013); (**b**) after the water temperature changing point (20 October 2013).

As shown in Figure 7b, 20 October 2013 represents the point of water temperature change in the East Sea of Korea. The distribution of the top 10 fish species was analyzed based on this time point. Before the water temperature change, species such as *Pleurnectidae* spp., *Oregoniidae* spp., *Octopodidae* spp., and *Buccinidae* spp. were included in the top 10 most frequently caught species (Figure 8a). After the water temperature change, new species such as *Lophiidae* spp. and *Hexagrammidae* spp. were included in the top 10 species, while the relative proportion of species, such as *Muraenidae* spp., tended to decrease (Figure 8b).

**Figure 9:**
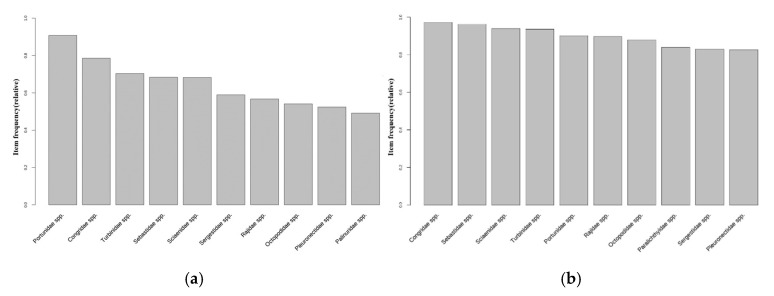
The frequency in which the top 10 species were caught in transactions in the West Sea: (**a**) before the water temperature change point (15 October 2013); (**b**) after the water temperature change point (15 October 2013).

In the West Sea, the date on which dominant species were caught before and after the water temperature fluctuation point was set to 15 October 2013 (Figure 7c). Following data analysis, *Portunidae* spp., *Congridae* spp., *Turbinidae* spp., *Sebastidae* spp., and *Sciaenidae* spp. were shown to be the top species before the water temperature fluctuation (Figure 9a). Even after the water temperature fluctuation, *Congridae* spp. and *Sebastidae* spp. remained among the top species, and benthic species such as *Sciaenidae* spp. were maintained (Figure 9b). However, the proportions of *Turbinidae* spp. and *Portunidae* spp. slightly decreased, while species including *Paralichthyidae* spp. and *Sergestidae* spp. entered the top ranks.

**Figure 10:**
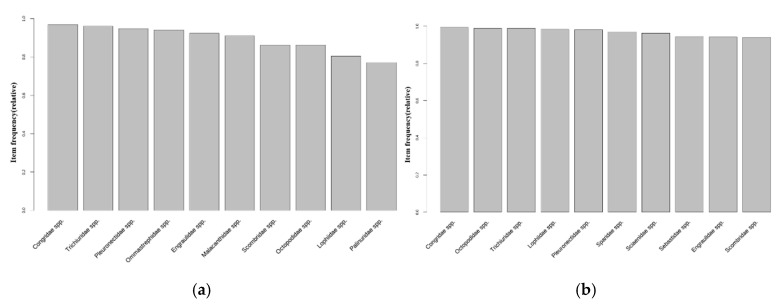
The frequency in which the top 10 species were caught in the transactions in the South Sea: (**a**) before the water temperature change point (25 February 2014); (**b**) after the water temperature change point (25 February 2014).

In the South Sea, the point in which the dominant species were caught before and after the water temperature fluctuation was set to 25 February 2014 (Figure 7d). An analysis of the data before the water temperature fluctuation indicated that *Congridae* spp., *Trichiuridae* spp., *Pleurnectidae* spp., *Ommastrephidae* spp., *Engraulidae* spp., and *Scombridae* spp. occupied the top ranks (Figure 10a). After the water temperature fluctuation, *Ommastrephidae* spp. and *Engraulidae* spp. remained among the top-ranked species, while *Lophiidae* spp., *Sparidae* spp., and *Sebastidae* spp. were newly included (Figure 10b).

### 3.4. Spatial Distribution of Key Species

As shown in Figure 6a, *Octopodidae* spp. was identified as one of the major fishery resources in Korea at the family level. However, this family includes species such as *Octopus minor*, *Enteroctopus dofleini*, and *Amphioctopus fangsiao*, whose spatial patterns could not be clearly identified at the species level.

**Figure 11:**
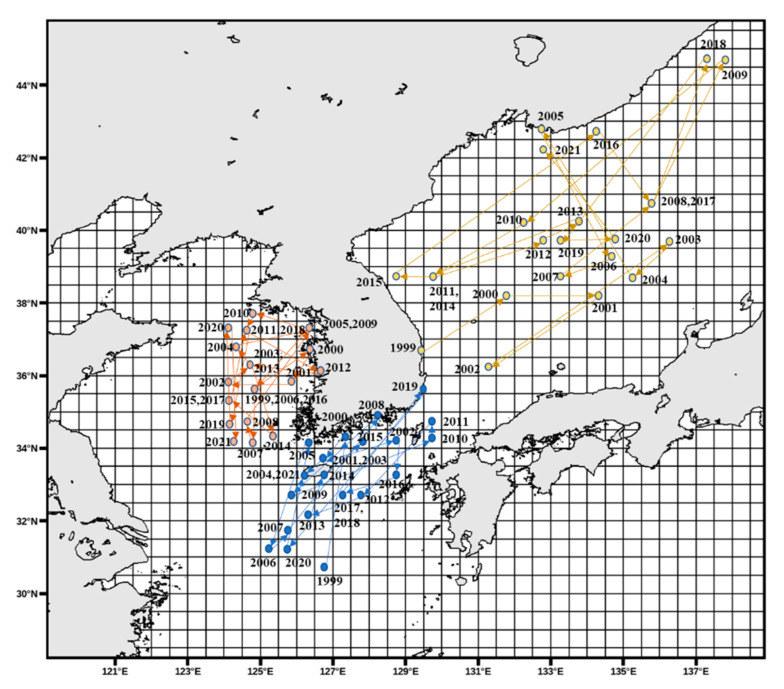
Spatial shifts in the CPUE centroids of *Todarodes pacificus* in the East, West, and South Seas from 1999 to 2021. These changes reflect regional variations in fishing activity and catch density. The orange, yellow, and blue arrows represent the directional movement patterns of CPUE centroids in the West Sea, East Sea, and South Sea, respectively.

Figure 11 illustrates that in the East Sea, *Todarodes pacificus* was more frequently observed along the southern coastal waters between 1999 and 2005. However, from 2016 onward, its distribution exhibited an overall northward expansion. In the West Sea, CPUE was generally the highest in the central region; however, compared to earlier years, the spatial concentration appeared to shift from northern to more southern waters. In the South Sea, the distribution was initially concentrated in the southeastern region, and later catch activity was observed to expand toward the waters south of Jeju Island.

**Figure 12:**
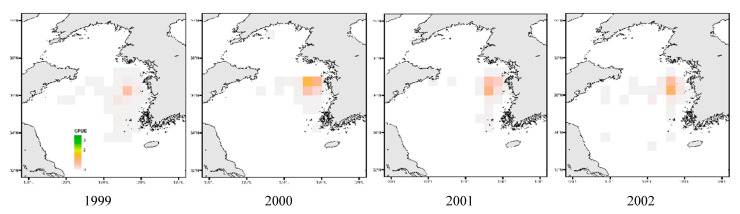

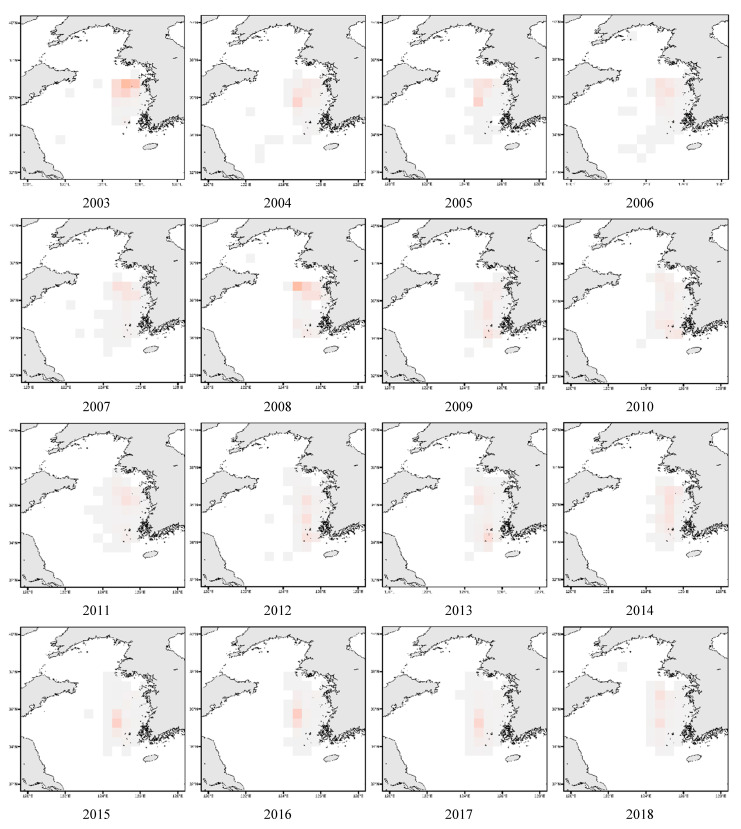

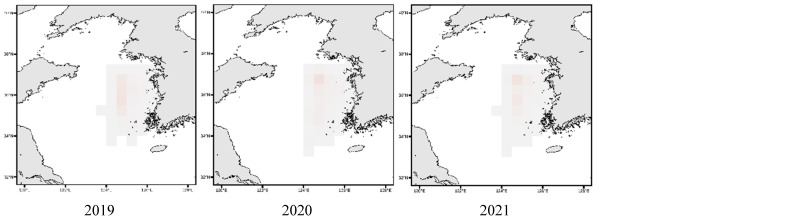
Distribution patterns of *Todarodes pacificus* catch per unit effort in West Sea from 1999 to 2021.

**Figure 13:**
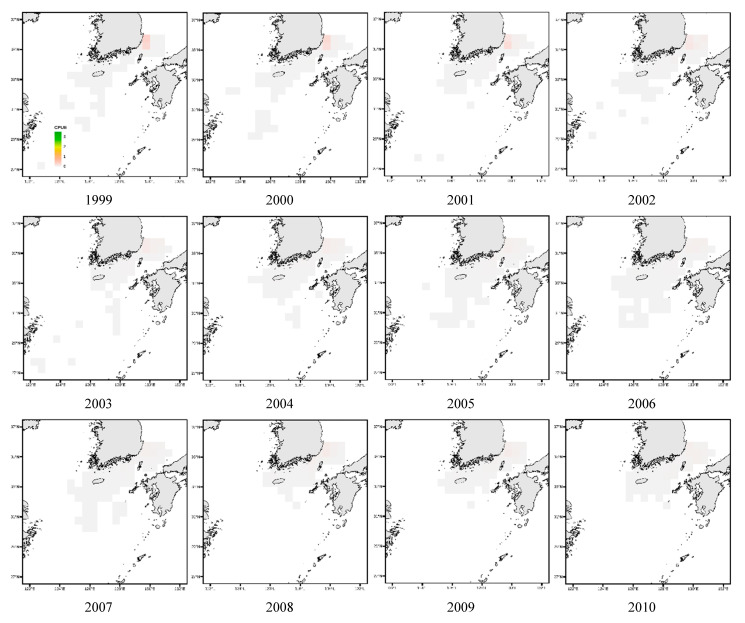

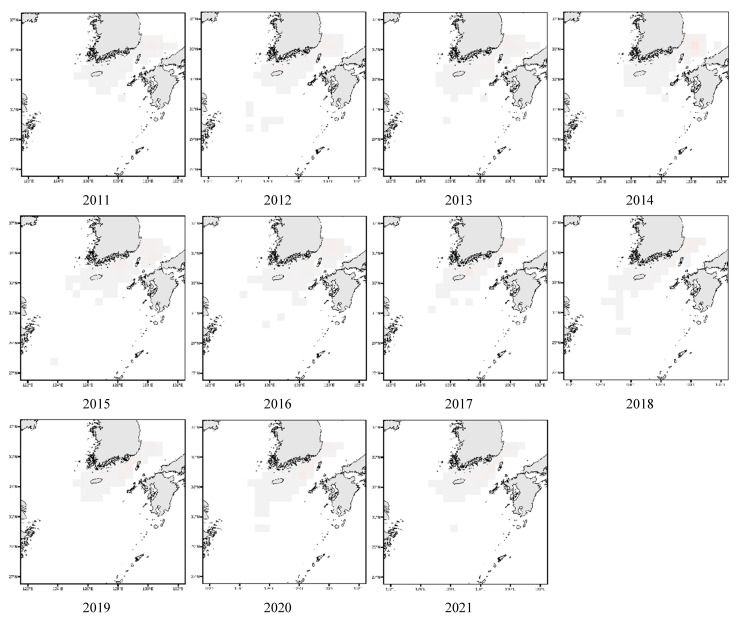
Distribution patterns of *Todarodes pacificus* catch per unit effort in South Sea from 1999 to 2021.

Figure 12 and Figure 13 depict the annual spatial variation in CPUE for *Todarodes pacificus*. In the East Sea, CPUE values were too low to represent a distinct spatial pattern. In the West Sea, as shown in Figure 12, the CPUE distribution pattern from 1999 to the late 2000s indicated relatively high values around latitude 34–37° N and longitude 124–126° E. In the South Sea, the distribution was initially concentrated in the southeastern region, and later catch activity was observed to expand toward the waters south of Jeju Island (Figure 13).

Similarly, as seen in Figure 11, from 1999 to the early 2000s, CPUE density was concentrated around latitude 34–35° N and longitude 129–130° E, although it remained lower than that of the West Sea. Additionally, the *Todarodes pacificus* fishing grounds in the South Sea showed a gradual contraction toward coastal waters.

## 4. Discussion

This study analyzed the spatiotemporal changes in catch frequency and species diversity in the waters around the Korean Peninsula from 1999 to 2021 and examined changes in dominant catch species according to changes in water temperature. To summarize these results, the catch frequency in the Korean Peninsula is increasing, and the analysis of species diversity (H-index) and caught species in the East Sea, West Sea, and South Sea of Korea from 1999 to 2021 showed an overall upward trend in the H-index. In addition, changes in water temperature were found to affect the composition of the dominant catch species in each water area. An analysis of changes in the fishing grounds of *Todarodes pacificus*, a commercially valuable species [23] that has declined by 30–40% according to the H-index analysis, revealed regional differences. However, overall, the CPUE density of *Todarodes pacificus* around Korea has decreased, with a reduction in its distribution range and irregular shifts in the central fishing grounds (Figure 11, Figure 12 and Figure 13).

First, according to the catch frequency analysis (Table 1 and Table 2), the catch frequency has increased rapidly since 2011, but the number of fishing zones has mostly remained the same. This suggests that the concentration of existing fishing zones has increased, and fishing activities have been carried out in more concentrated areas. McConnaughey et al. [24] indicated that although various factors such as the design of fishing gear work in a complex manner if the frequency and intensity of fishing activities (e.g., trawls) are concentrated, it may make it difficult to recover the long-term ecosystem and promote decreases in fishery productivity. Moreover, fishing intensity has a significant impact on the trophic structure of the ecosystem [25]. As fishing intensity increases, fish species at higher trophic levels may become closer to extinction, which may lead to significant changes in the structure of the entire ecosystem [25]. In particular, in the case of non-selective fishing, the higher the fishing intensity, the more likely it is that fish species at higher trophic levels will become extinct first, leaving only fish species at the bottom of the food chain. Thus, total production can be maintained at a high level, but the ecosystem structure is seriously damaged [25]. The concentration of fishing activities in specific marine areas can have long-term impacts on the marine ecosystem. Therefore, it is essential to implement fisheries management policies that regulate fishing intensity in certain areas and strengthen ecosystem protection. In particular, in response to biodiversity changes caused by factors such as climate and environmental changes, the number and extent of coastal and marine protected areas (MPAs) have been expanded. These protective measures have emerged as one of the most effective strategies for conserving biodiversity and its resources [26].

When analyzing the species diversity in the waters around the Korean Peninsula between 1999 and 2021, an upward trend in diversity was observed rather than a sharp decrease. However, as shown in Figure 7, the water temperature in the waters around the Korean Peninsula was found to have been rising from a certain point in time, which indicates that the marine ecosystem environment is changing. According to Poloczanska et al. [27], this increase in water temperature can be a major factor in changing the ecosystem structure by causing species to move their habitats. As some species expand to new areas due to climate change, the competitive landscape within the existing ecosystem can be reorganized, and new ecological niches can be formed. Subsequently, species diversity may have temporarily increased in the waters around the Korean Peninsula due to the introduction of new species and increased ecological opportunities. The reason for this evaluation is that while an improvement in ecosystem resilience can increase biodiversity, according to an analysis of a study that did not evaluate the biodiversity of the surrounding waters, the trophic level of the marine ecosystem as an indicator of biodiversity change revealed a decrease in the waters around the Korean Peninsula from 1971 to 2020. In particular, the decreasing trend of the trophic level in the West Sea is more evident than in other waters; however, this level is also estimated to be relatively low on average [7].

In the East Sea, the change in species diversity was relatively stable following the change in water temperature; however, the variability in species diversity increased in the South and West Seas (Figure 3). This can be seen by examining the characteristics of these sea areas. The East Sea is relatively less affected by changes in water temperature due to its deep water depth and geographical characteristics. However, the tidal difference is large in the West Sea. In winter, most of the water is mixed to the bottom [28], meaning the atmospheric temperature greatly affects the water temperature. The South Sea has characteristics that are different from those of the East and West Seas, and it is judged that the characteristics of the change in species diversity reflect these characteristics. In addition, when looking at the spatial change pattern of species diversity, most of the waters closer to the coast were greener and showed high species diversity. The distribution range in the East Sea was limited to the northern and central seas and did not tend to spread to the south (Figure 5). In the West Sea, it spread to the north and south, and in the South Sea, the species diversity in the southwestern sea area tended to decrease (Figure 5). Climate change is a major factor in the change in species distribution [29]. Thus, conditions generally become more suitable toward the poles and less suitable toward the equator [30]. Indeed, Ju and Kim [31] confirmed the northward movement of subtropical species in the South Sea. Moreover, Ju and Kim [32] confirmed the northward movement of subtropical species in the waters near Jeju Island.

Furthermore, Dulvy et al. [33] observed that fish species shifted to deeper waters as sea temperatures rapidly increased in the North Sea, suggesting that biological responses to temperature changes may follow similar global patterns. Northward shifts and horizontal expansions have been observed in the South and West Seas, likely driven by environmental factors within the ecosystem. The habitat ranges of fish species appear to have shifted based on their ecological characteristics, such as eurythermal or stenothermal traits. Specifically, the latitudinal response to warming was heterogeneous, reflecting (i) a northward shift in the mean latitude of abundant, widespread thermal specialists and (ii) a southward shift in relatively small, abundant southern species with limited ranges and a northern boundary in the North Sea [34]. In this study, the trend line analysis results show that the timing of the water temperature change varied depending on the sea area but generally occurred between 2013 and 2014 (Figure 7). However, spatiotemporal changes in species diversity were continuously observed before and after this point. Dulvy et al. [33] analyzed the 1 °C water temperature increase between 1988 and 1989, which caused significant changes in the geographical distribution and the behavior of benthic fish populations in the North Sea. As a result of the rise in water temperature, benthic fish populations moved to deeper waters by an average of about 3.6 m per 10 years, with this phenomenon being particularly prominent between the late 1980s and the mid-1990s. This study also showed similar results for the impact of climate change, which continuously affected the reorganization of the marine ecosystem structure before and after the point of the water temperature change.

This study analyzed the changes in major fish species caught in the waters around Korea from 1999 to 2021 and analyzed the changes in the distribution and dominance of fish species according to the time of the water temperature change. As a result, it was confirmed that the water temperature change at a specific time (Figure 7b–d) significantly impacted the distribution and ecological characteristics of each fish species. In the East Sea, West Sea, and South Sea, changes occurred in the composition of the top fish species after the water temperature change, suggesting that the marine environment change related to climate change is a major factor in reorganizing the structure of the aquatic ecosystem. In particular, when a comparative analysis was performed based on the water temperature change point, the species that consistently dominated the catch in the East Sea, West Sea, and South Sea from 1999 to 2021 were *Octopodidae* spp. and *Ommastrephidae* spp. These species (cephalopods) generally display a semelparous life history strategy, with a life span of about 1 year [34]. Somero [35] found that, similar to species that are less affected by climate change and whose resources are maintained to ‘Winners’, these species have greater genetic diversity and adaptability, such as those with shorter generation times and broader thermal tolerances, and they are more likely to survive climate change. Furthermore, benthic fish such as *Pleuronectidae* spp. also almost constantly maintain their fishing dominance, and benthic fish species are less affected by climate change. According to Rubin [36], the distribution and population of benthic fish species reflect the average conditions of the ocean and are relatively slow to respond to environmental changes. However, this suggests that benthic fish species are more likely to be affected by long-term and continuous changes than short-term climate fluctuations [36]. Conversely, compared to pelagic fish species (pelagic fish species), benthic fish species have adapted to constant environmental conditions over a longer period, meaning they may be less likely to experience immediate distribution changes due to climate change [36].

Among the dominant species of Ommastrephidae, *Todarodes pacificus* exhibited spatiotemporal variations in its distribution patterns, with continuous declines in both the central fishing grounds and CPUE density over the years. Similarly, the study by Kim et al. [37] reported spatial CPUE variation patterns consistent with the findings of this study. In the West Sea, fishing grounds were generally concentrated in adjacent areas during the 1990s, while in the East Sea, the central fishing grounds were located at the highest latitudes in the 1980s (similar to the patterns shown in Figure 11). In the 1990s, the fishing grounds shifted southward, corresponding to the South Sea region in this study (Figure 13), and by the 2000s, the fishing grounds were found closest to the coast. This shift was attributed to changes in the Kuroshio Current pathway [38].

The variation in the Kuroshio Current pathway can be explained by the Pacific Decadal Oscillation (PDO). According to Kim [38], the PDO is significantly correlated with the catch volume of *Todarodes pacificus* in the East Sea. When the PDO is in a negative (−) phase, the inflow of the Kuroshio Current into the East Sea increases, strengthening the current and resulting in fishing grounds forming closer to the coast. Conversely, during a positive (+) PDO phase, the inflow decreases, weakening the current and pushing the fishing grounds further offshore. These changes affect fishing accessibility and economic profitability, ultimately influencing catch volume.

The commercial value and fluctuations in the stock of *Todarodes pacificus* have raised concerns about the necessity of resource management. As a response, minimum catch size regulations and closed fishing seasons were implemented. However, the effectiveness of these management measures remains uncertain, as the total allowable catch exhaustion rate for fisheries operating under these regulations remained at around 30% for three consecutive years (2018–2020) [23], underscoring the need for more effective resource management strategies. As mentioned earlier, MPAs have been recognized as one of the most effective measures for biodiversity conservation and resource preservation. However, when MPAs are established at different times across various regions, their effectiveness may be limited. To achieve more effective protection, it has been suggested that MPAs should extend beyond national jurisdiction [26]. The findings of this study highlight the need to identify seasonally or regionally sensitive species in order to prioritize fisheries resource management. Furthermore, developing region-specific management strategies that account for environmental sensitivity and species-specific ecological traits is essential for effective and adaptive resource governance.

This study employed a frequency-based analysis to calculate species diversity. While this approach provides useful insights, it is important to acknowledge that diversity indices calculated from catch volume data are inherently limited as they may be biased by market preferences and regulatory factors. Additionally, the findings of this study primarily consider water temperature as the main factor influencing species diversity, which presents a clear limitation as it does not fully reflect the various other factors contributing to biodiversity changes. Future research should incorporate additional environmental variables, such as salinity, dissolved oxygen, chlorophyll-a, and ocean currents to better explain their interrelationships and impacts on species diversity. In particular, while the Prophet model used in this study is well suited for detecting temporal patterns, future studies should consider applying Generalized Additive Models (GAMs) or Generalized Linear Models (GLMs) to more effectively analyze the relationships between environmental factors (e.g., water temperature, salinity, and dissolved oxygen) and species diversity.

## 5. Conclusions

This study analyzed catch data and water temperature changes in Korean waters from 1999 to 2021 to evaluate the ecological impacts of climate change. The results show region-specific shifts in species composition, with the West and South Seas exhibiting greater variability than the East Sea. Species such as *Octopodidae* spp. and *Ommastrephidae* spp. remained dominant, indicating potential resilience, whereas *Todarodes pacificus* showed notable declines, highlighting the vulnerability of certain commercial species to warming trends. These findings support the study’s objective of identifying spatiotemporal trends in catch composition under changing oceanographic conditions. They also emphasize the need for adaptive resource management strategies, such as refining size limits, revising fishing seasons, and implementing spatial protections. While this study primarily focused on sea temperature, we recognize the need to include other environmental variables such as salinity, dissolved oxygen, and chlorophyll-a to better understand their combined influence on marine biodiversity. Future studies should apply multifactorial approaches using models such as GAMs or GLMs to strengthen ecological interpretation and inform ecosystem-based fisheries management.

## Figures and Tables

**Table 1:** Structure and description of Catching Activity Record data.

Variable	Explanation
Date	The total number of days for which operational data were reported to the Safety Operation Headquarters was 8248.
Fishing method	There are 88 types of coastal industries in Korea.
Sea	The fishing areas include the East Sea, South Sea, West Sea, and other sea areas including the Pacific Ocean below Japan. The division of sea areas in the data is based on Figure 1 for consistency.
Zone	This refers to the ocean basin; meanwhile, a small zone is a small area divided into nine ocean basins. In this study, the ocean basin was analyzed.
Fish name	The name of the species of fish caught.
Quantity	The catch volume, reported by fishermans, is based on specific spatial and temporal data, and the unit is kilograms (kg).

**Table 2:** The annual frequency of fishing operations from 1999 to 2021 in the East, West, and South Seas, as well as other maritime regions.

Year	Number of Fishing Days	Fishing Frequency	Number of Trenches Where Fishing Activities Took Place (Sea)			
			East	South	West	ETC
1999	214	80,259	69	108	68	4
2000	365	142,957	73	139	78	5
2001	365	192,517	86	156	79	2
2002	364	212,506	95	170	74	6
2003	365	282,699	119	167	74	1
2004	366	345,548	129	158	77	0
2005	365	345,291	153	156	76	0
2006	365	403,085	138	147	80	0
2007	365	420,316	138	143	71	0
2008	366	364,140	125	146	70	0
2009	365	320,661	140	142	62	0
2010	365	305,918	139	150	68	3
2011	365	314,866	144	149	66	1
2012	366	467,570	135	147	62	0
2013	365	896,084	135	151	67	1
2014	365	969,692	140	138	67	0
2015	365	861,863	135	153	65	0
2016	366	810,794	142	156	70	0
2017	365	871,717	137	111	67	0
2018	365	1,051,979	144	129	68	1
2019	365	1,347,303	148	125	58	1
2020	366	1,354,784	113	128	66	0
2021	365	1,264,167	120	122	60	0

**Table 3:** The monthly frequency of fishing operations from 1999 to 2021 in the East, West, and South Seas, as well as other maritime regions.

Month	Fishing Frequency	Number of Trenches Where Fishing Activities Took Place (Sea)			
		East	South	West	ETC
1	866,985	86	165	81	1
2	672,618	88	171	83	2
3	891,609	86	191	83	2
4	977,095	84	183	84	1
5	1,211,875	88	171	85	1
6	1,279,220	87	154	84	3
7	1,169,302	157	126	79	2
8	1,232,525	179	117	86	4
9	1,429,914	175	115	85	2
10	1,483,153	152	143	86	5
11	1,302,713	95	150	85	1
12	1,109,707	92	157	86	0

## Data Availability

All data and materials are available and can be provided to the journal upon reasonable request.

## Abbreviations

The following abbreviations are used in this manuscript:

CAR	Catching Activity Record
KODC	Korea Oceanographic Data Center
IQR	InterQuartile Range
CPUE	Catch Per Unit Effort
MPAs	Marine Protected Areas
PDO	Pacific Decadal Oscillation
GAMs	Generalized Additive Models
GLMs	Generalized Linear Models
